# Assessing national and subnational inequalities in medical care utilization and financial risk protection in Rwanda

**DOI:** 10.1186/s12939-019-0953-y

**Published:** 2019-03-27

**Authors:** Kai Liu, S. V. Subramanian, Chunling Lu

**Affiliations:** 1 Department of Social Security, School of Labor and Human Resources, Renmin University of China, Beijing, China; 2 Department of Social and Behavioral Sciences, Harvard T.H. Chan School of Public Health, Boston, MA USA; 3 Division of Global Health Equity, Brigham & Women’s Hospital, Boston, MA USA; 4 Department of Global Health and Social Medicine, Harvard Medical School, Boston, MA USA; 5 Department of Science and Technology-National Research Foundation (DST-NRF) Center of Excellence in Human Development, University of Witwatersrand, Johannesburg, South Africa

**Keywords:** Health inequality, Medical care utilization, Household catastrophic health spending, Absolute inequality, Relative inequality, Rwanda

## Abstract

**Background:**

Ensuring equitable access to medical care with financial risk protection has been at the center of achieving universal health coverage. In this paper, we assess the levels and trends of inequalities in medical care utilization and household catastrophic health spending (HCHS) at the national and sub-national levels in Rwanda.

**Methods:**

Using the Rwanda Integrated Living Conditions Surveys of 2005, 2010, 2014, and 2016, we applied multivariable logit models to generate the levels and trends of adjusted inequalities in medical care utilization and HCHS across the four survey years by four socio-demographic dimensions: poverty, gender, education, and residence. We measured the national- and district-level inequalities in both absolute and relative terms.

**Results:**

At the national level, after controlling for other factors, we found significant inequalities in medical care utilization by poverty and education and -in HCHS by poverty in all four years. From 2005 to 2016, inequalities in medical care utilization by the four dimensions did not change significantly, while the inequality in HCHS by poverty was reduced significantly. At the district level, inequalities in both medical care utilization and HCHS were larger than zero in all four years and decreased over time.

**Conclusions:**

Poverty and poor education were significant contributors to inequalities in medical care utilization and HCHS in Rwanda. Policies or interventions targeting poor households or households headed by persons receiving no education are needed in order to effectively reduce inequalities in medical care utilization and HCHS.

**Electronic supplementary material:**

The online version of this article (10.1186/s12939-019-0953-y) contains supplementary material, which is available to authorized users.

## Background

An important aspect of the Sustainable Development Goals (SDGs) is to eliminate within-country health inequities [1, 2]. Ensuring access to care and providing financial risk protection have been the goals of government policies or programs in many developing countries (e.g. China, Ghana, Mali, and Mexico, Rwanda, and Vietnam) [3–9]. A core essence of health equity lies in mitigating systematic disparities in medical care utilization and household catastrophic health spending (HCHS) in different social groups [10–12]. To monitor and evaluate the effects of interventions on reducing health inequalities and make comparative studies across countries or over time, it is crucial to assess the levels and trends of inequalities in medical care utilization and HCHS using various measures.

Rwanda is a low-income country in eastern Africa with a population of 11.6 million in 2015. Approximately 83% of the population live in rural areas [13]. Though Rwanda has been making remarkable progress in both social and economic development, as evidenced by its increasing GDP per capita from US$349 (constant 2010 US$) in 2001 to US$690 in 2015 [13], it remains one of the most undeveloped countries in the world, with 39.1% of its citizens living below the national poverty line in 2014 [14].

To promote health equity and universal health coverage, the Government of Rwanda took a series of actions, including: piloting *Mutuelles*, a community-based health insurance program; developing health facilities; changing payment methods for healthcare providers; and so on [15, 16]. Ensuring equitable access to medical care with financial risk protection has been at the center of achieving universal health coverage [17–23]. Therefore, this study focuses on medical care utilization among those in need and HCHS resulting from seeking medical care.

Our previous studies in rural Rwanda have found that, from 2005 to 2010, significant inequalities persisted between the poor and non-poor households in medical care utilization or HCHS [17], and that the inequalities in medical care utilization and HCHS between the poverty and non-poverty groups in Rwanda were not explained merely by the difference in their economic status; other factors, including *Mutuelles* status and geographic access to health care facilities also contributed significantly to the inequalities by poverty [24]. This study extends the previous studies by quantifying health inequalities in various socio-demographic dimensions. Using the Rwanda Integrated Living Conditions Survey (EICV) in 2005, 2010, 2014, and 2016, we assessed the levels and trends of the inequalities of these two health indicators by four dimensions: poverty status, gender, education, and place of residence. In addition, we also investigated the sub-national inequalities, as Rwanda has decentralized health care to the districts and the country’s interventions for reducing health inequalities have been organized and carried out at the district level [18].

## Methods

### Data and sample

We used data from the repeated cross-sectional EICV surveys of 2005, 2010, 2014, and 2016 for our analysis [25]. The EICV surveys provide information about household income and expenditure, household and individual’s demographic and socioeconomic characteristics, health insurance status, medical care utilization and related out-of-pocket health spending (OOPS). Details on its sampling and implementation processes are presented in Additional file 1: Box S1.

To estimate the levels and trends of inequalities in medical care utilization, we used the individual as the unit of analysis and included in our sample only those who reported being ill for two or four weeks before each survey. The final sample size was: 6737 in 2005; 11,944 in 2010; 16,807 in 2014; and 21,150 in 2016. To estimate the levels and trends of the inequalities in HCHS, we used the household as the unit of analysis and included all of the households in the 4 years, and the final sample size was: 6639 in 2005; 11,335 in 2010; 14,125 in 2014; and 14,548 in 2016.

### Variables

#### Medical care utilization among those who reported being ill and related variables

A dichotomous outcome variable was constructed to indicate medical care utilization among individuals who reported being ill in the previous two weeks of the EICV 2 (2005) and EICV 3 (2010) surveys, and in the previous four weeks of the EICV 4 (2014) and EICV 5 (2016) surveys. Medical consultation is the only variable that was available across all four surveys. To make sensible comparisons, medical services in this study includes only medical consultation in hospitals or health centers.

Using the information available in each EICV, we constructed four dichotomous variables indicating if an individual who reported being ill was either female, had a household head without schooling, resided in a rural area, or lived below the national poverty line. Because children under the age of 18 typically rely on their care-givers for care-seeking decision making, we used the schooling level of the head of each household in our analysis. The EICV surveys have a variable indicating a household’s poverty status. The poverty status was defined as at 64,000 Rwanda Francis (US$144.47) per adult per year in January 2001 prices in the EICV 2 (2005) and EICV 3 (2010) data [26], and 159,375 Rwanda Francis (US$233.35) in January 2014 prices in the EICV 4 (2014) and EICV 5 (2016) data [27]. We adjusted for health care needs, geographic access to medical care, individual health insurance status, and household size. Health care needs were represented by two variables: a categorical age variable (under 30 years of age, between age 30 and 50, and over 50 years of age), and a dummy variable “having disability” denoting whether or not an individual had a disability. To measure geographic access to medical care, we constructed a dummy variable, “travel time to health center,” to represent travel time of more than 0.5 h to the nearest health center. A dichotomous variable was constructed to indicate health insurance status. Summary statistics for these variables are presented in Additional file 1: Table S1.

#### HCHS and related variables

We constructed a dichotomous outcome variable indicating whether or not a household had catastrophic health expenditure, defined as annual OOPS exceeding 40% of the household’s annual capacity to pay [28, 29]. Our previous study has demonstrated that estimation of OOPS is sensitive to the recall period, number of questions and design of the survey modules [30]. To avoid confounding effects from the survey’s design, we used the same OOPS information in the consumption module with a recall period of 1 year in all of the four survey years. Details on obtaining HCHS are presented in the Additional file 1: Box S2.

We constructed four dichotomous variables to indicate gender, the schooling level achieved by the head of a household, residence, and the poverty status of a household. Health need is measured with a series of categorical variables that indicate if a household had under-five children or any disabled members. Other covariates included the age of the head of the household, travel time to the nearest health center, household health insurance status and household size. Summary statistics of these variables are presented in the Additional file 1: Table S2.

### Statistical analysis

Our objective in this research is to assess the inequalities in the two health indicators with four dimensions (i.e., poverty, gender, education and residence) at the national level and derive a summary measure of inequalities at the district level. Following the research of Harper [31–34], we used absolute and relative inequality measures to estimate the inequalities. Absolute inequality was measured by the difference in the percentage of individuals using medical care when in need or the percentage of households incurring HCHS between our two social groups (e.g. non-poverty and poverty). Higher absolute values of the absolute inequalities indicate a high level of inequality; zero implies no inequalities. Relative inequality was measured by the ratio of the percentage of medical care utilization or the percentage of HCHS between the two social groups. The relative inequalities moving away from 1 indicate a high level of inequality with 1 indicating no inequality. These inequality measures have been commonly used in empirical analyses and are easy to communicate to policy makers and other non-technical readers. In this study, we mainly report the estimates of the absolute inequality in the text and we used the estimates of the relative inequality as a sensitivity test.

The methods for estimating inequalities in medical care utilization and HCHS in previous studies have been inconsistent. Some publications used the unadjusted differences of the two health indicators between population groups [35–40]. Other studies defined inequality as the differences between the population groups after adjusting for factors such as health needs or socio-economic characteristics [17, 24, 41–45]. Risk adjustment could address the confounding influence of these variables and enable us to have a better understanding about the components of inequalities in order to make well-targeted policies. Therefore, we adopted adjusted inequalities.

#### Measuring adjusted inequalities of the two health indicators

At the national level, we conducted logistic regression analysis, which allowed us to obtain the adjusted mean (i.e. the marginal effect) of using medical care utilization and incurring HCHS between the two social groups (e.g. non-poverty vs. poverty) in the four survey years, controlling for other factors (see Eq. 1 below**)**. Taking poverty status as an example, we processed our analysis with data from each of the four survey years.

1 $$ Logit\left({Utilization}_i/{HCHS}_i\right)={\beta}_0+{\beta}_1{Poverty}_i+\boldsymbol{\beta} {\boldsymbol{X}}_{\boldsymbol{i}} $$

where Logit(Utilization_i_/HCHS_i_) represents the probability of using medical care or incurring HCHS for the *i*^*th*^ individual or household, and Poverty_i_ is the poverty status of the *i*^th^ individual or household. ***β*** is a vector of the coefficients for **X**_**i**_ that is a vector of variables on individual or household health needs, insurance status, geographic difficulty in accessing care, and other socioeconomic variables including gender, education and place of residence.

Using the marginal effects of covariates, we were able to derive the adjusted mean likelihood, and their 95% confidence intervals (CIs) of using medical care and incurring HCHS in the four survey years for each social group.

At the district level, we first calculated the adjusted percentage of individuals using medical care when in need and the adjusted percentage of households incurring HCHS for each of the 30 districts in Rwanda in the four survey years. District-level inequalities were then estimated with two complex measures: (1) the absolute weighted mean from the national mean and (2) the relative weighted mean from the national mean [46]. The former was calculated as the sum of the absolute values of the mean difference between each district and the national average, weighted by each district’s population share^1^. The latter was calculated as the absolute weighted mean divided by the national average^2^. Both measures take only positive values, with larger values indicating higher level of inequality and 0 indicating no inequality (Table 1).

**Table 1:** Summary of health inequality measures at the national and district level

	Medical care utilization		Catastrophic health spending	
	Absolute inequality	Relative inequality	Absolute inequality	Relative inequality
National-level (by poverty, gender, education and residence)	Adjusted absolute inequality (mean difference)	Adjusted relative inequality (mean ratio)	Adjusted absolute inequality (mean difference)	Adjusted relative inequality (mean ratio)
District-level	Adjusted absolute weighted mean difference from the overall mean	Adjusted relative weighted mean difference from the overall mean	Adjusted absolute weighted mean difference from the overall mean	Adjusted relative weighted mean difference from the overall mean

#### Testing the statistical significance of the levels and trends of the inequalities

To test whether inequalities in medical care utilization or HCHS significantly existed in the four survey years, we examined whether the 95% CIs of the absolute estimates crossed the value of zero and whether the relative estimates crossed the value of one.

To determine whether inequalities in medical care utilization or HCHS significantly changed from 2005 to 2016, we compared the 95% CIs of the two estimates using Cumming and Finch’s “rule of thumb” for comparing two independent means [47]. We calculated three components that were required to implement the “rule of thumb”: marginal errors, overlap of the 95% CIs of the two estimates, and proportion overlap. Marginal error refers to the absolute difference between the mean and its lower or higher bound of a 95% CI. An overlap of CIs refers to the difference between one estimate’s higher bound and the other estimate’s lower bound. Proportion overlap is then calculated as the overlap of the 95% CIs between the two estimates divided by the average marginal errors between the two estimates. According to the “rule of thumb”, when sample sizes of two samples are both at least 10, and the margins of error between the two estimates do not differ by more than a factor of two, a proportion overlap less than 0.5 indicates a significant statistical relationship at the 0.05 level (i.e. *p* < 0.05). An example of and the formulas we used for these calculations are presented in Table 3 and Additional file 1: Box S3, respectively.

We used Stata 14.0 for all of our analyses.

## Results

### Inequalities in medical care utilization between social groups and at the district level

**Fig. 1:**
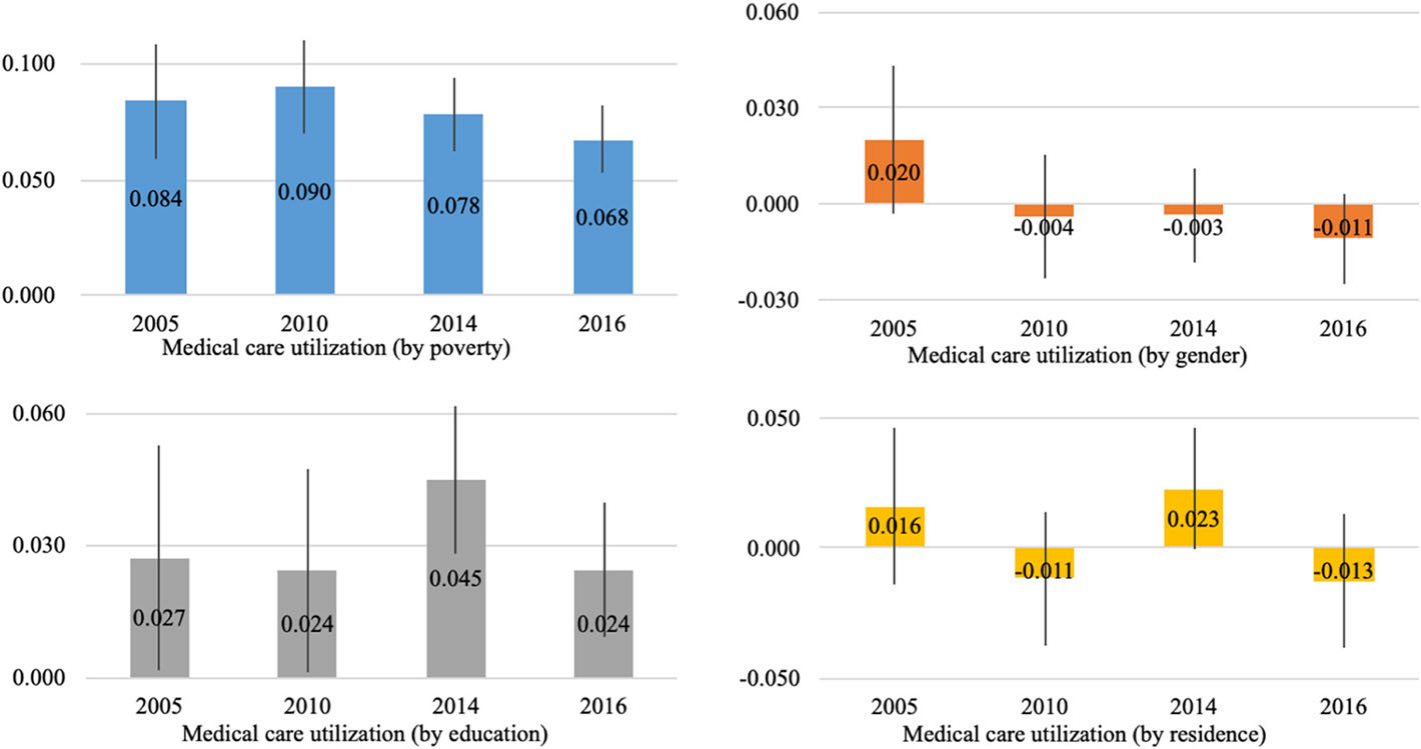
The adjusted absolute inequality in medical care utilization by the status of poverty, gender, education and residence

In the four survey years under investigation, the absolute inequalities by poverty (0.084 in 2005, 0.090 in 2010, 0.078 in 2014 and 0.068 in 2016) and education (0.027 in 2005, 0.024 in 2010, 0.045 in 2014 and 0.024 in 2016) in medical care utilization were significantly larger than zero (Fig. 1). The results remained unchanged when using the relative inequality measures (Additional file 1: Figure S1).

From 2005 to 2016, though there were changes in the absolute inequality by the four dimensions, the Cumming and Finch tests show that none of these changes was statistically significant, except for the inequality by gender that was significantly reduced (Table 2). The results remained unchanged when using the relative inequality measures (Additional file 1: Table S3).

**Table 2:** Significance testing of the trends of the absolute inequality in medical care utilization from 2005 to2016 using Cumming and Finch’s “rule of thumb”

	2005 (*N* = 6737)				2010 (*N* = 11,944)				2014 (*N* = 16,807)				2016 (*N* = 21,150)				Proportion overlap			
	Mean	LB	HB	ME	Mean	LB	HB	ME	Mean	LB	HB	ME	Mean	LB	HB	ME	2005 vs 2010	2010 vs 2014	2014 vs 2016	2005 vs 2016
Poverty	0.084	0.059	0.109	0.025	0.090	0.070	0.110	0.020	0.078	0.062	0.094	0.016	0.068	0.053	0.082	0.015	1.735	1.350	1.301	1.166
Gender	0.020	−0.003	0.043	0.023	−0.004	−0.023	0.016	0.019	− 0.003	− 0.018	0.012	0.015	− 0.011	− 0.025	0.004	0.014	0.874	1.977	1.489	0.339
Education	0.027	0.002	0.053	0.026	0.024	0.001	0.048	0.023	0.045	0.028	0.062	0.017	0.024	0.009	0.040	0.016	1.888	0.968	0.716	1.848
Residence	0.016	−0.014	0.046	0.030	−0.011	− 0.037	0.014	0.026	0.023	−0.001	0.046	0.023	−0.013	−0.038	0.013	0.026	1.008	0.620	0.571	0.963

Note: N is the number of observations, LB is the lower bound of the 95% CIs, HB is the higher bound of the 95% CIs, and ME refers to the margins of error. Margins of error is the distance of either the lower or the higher bound 95% CI from the mean. The proportion overlap is defined as the intervals overlap between the two independent samples, expressed as a proportion of the average margin of error. For example, in the adjusted inequality by poverty from 2005 to 2016 above, 1.166 = (0.082–0.059)/[(0.025 + 0.015)/2]. According to Cumming and Finch [45], when both sample sizes are at least 10, and the margins of error do not differ by more than a factor of two, a proportion overlap less than 0.5 indicates a significant statistical relationship at the 0.05 level (*p* < 0.05). Therefore, the absolute differences in the two years are not statistically significant

For the district-level measures, the adjusted absolute weighted mean difference from the overall mean was higher than zero in all of the four survey years (0.028 in 2005, 0.041 in 2010, 0.033 in 2014, and 0.020 in 2016) (Table 3). Over time, districts such as Nyanza and Nyamagabe had low percentage of medical care utilization, and Nyarugenge and Musanze had high percentage of medical care utilization. Percentage of utilization in the district with the lowest value in 2016 (49.9% in Nyanza) was higher than that in the district with the highest value in 2005 (42.8% in Nyarugenge) (Additional file 1: Table S7). The adjusted absolute weighted mean difference from the overall mean decreased by 26.98% from 2005 to 2016 (Table 3). The results remained unchanged when using the relative inequality measures (Additional file 1: Table S4). From 2005 to 2016, among the 30 districts, Nyaruguru (137.99%) experienced the largest increase in the adjusted level of medical care utilization, followed by the districts of Gisagara (119.60%) and Burera (110.25%). Nyarugenge (40.65%) had the smallest increase in the adjusted level of medical care utilization (Additional file 1: Table S7).

**Table 3:** The absolute weighted differences between the district and the national means for medical care utilization and HCHS

	2005 (*N* = 30)	2010 (*N* = 30)	2014 (*N* = 30)	2016 (*N* = 30)	Percent change from 2005 to 2016
Medical care utilization	0.028	0.041	0.033	0.020	−26.98%
HCHS	0.017	0.016	0.003	0.010	−38.74%

Note: N is the number of observations

### Inequalities in HCHS between social groups and at the district level

**Fig. 2:**
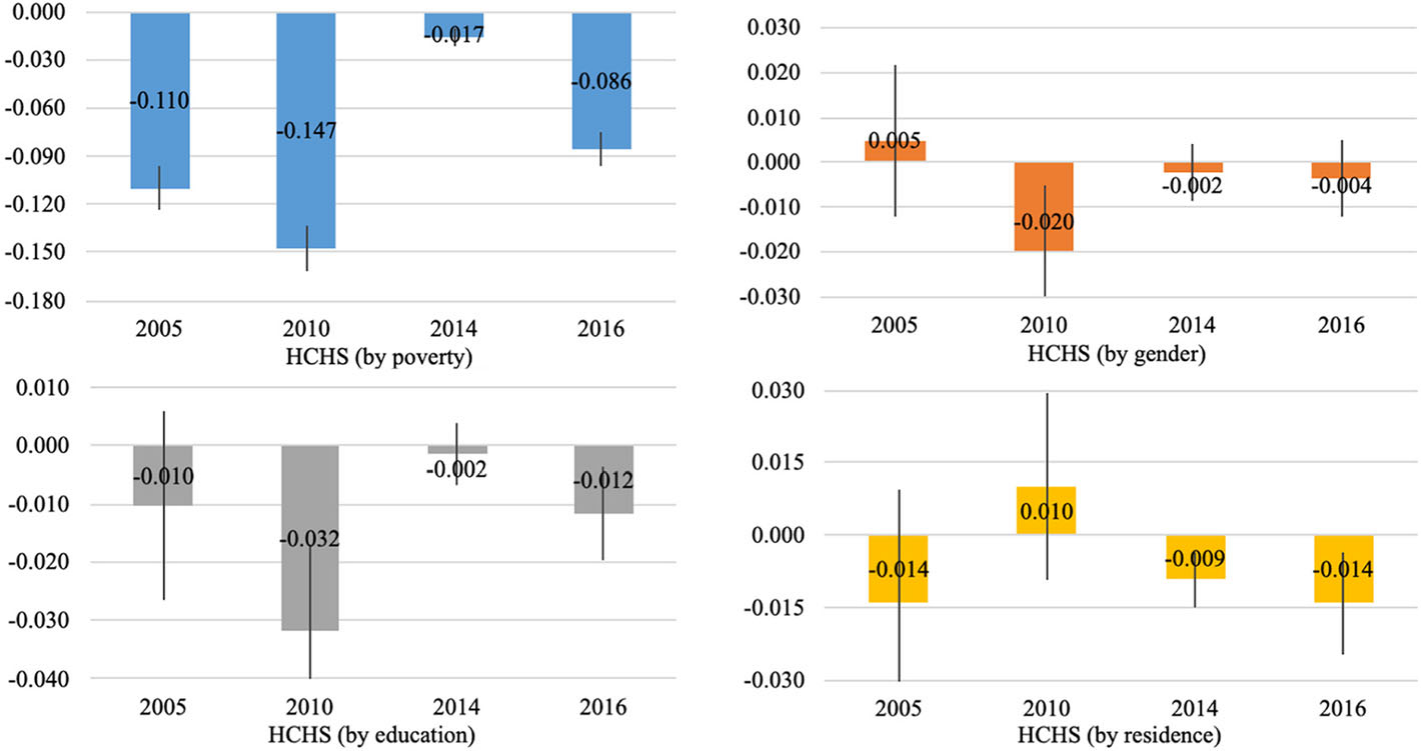
The adjusted absolute inequality in HCHS by the status of poverty, gender, education and residence

In the four survey years, the absolute inequalities by poverty (− 0.110 in 2005, − 0.147 in 2010, − 0.017 in 2014, and − 0.086 in 2016), gender (− 0.020 in 2010), education (− 0.032 in 2010 and − 0.012 in 2016), and residence (− 0.009 in 2014 and − 0.014 in 2016) in HCHS were significantly smaller than zero (Fig. 2). The results remained unchanged when using the relative inequality measures (Additional file 1: Figure S2).

From 2005 to 2016, the absolute inequalities by poverty status in HCHS significantly decreased. Although we observed a fall in the absolute inequalities by gender, this decline was not statistically significant according to the Cumming and Finch tests (Table 4)**.** When using the relative inequality measures, the national-level trends remained unchanged, with one exception: there was no significant change in the relative inequalities in HCHS by poverty from 2005 to 2016. (Additional file 1: Table S5).

**Table 4:** Significance testing of the trends of the absolute inequality in HCHS from 2005 to 2016 using Cumming and Finch’s “rule of thumb”

	2005 (*N* = 6639)				2010 (N = 11,335)				2014 (*N* = 14,125)				2016 (N = 14,548)				Proportion overlap			
	Mean	LB	HB	ME	Mean	LB	HB	ME	Mean	LB	HB	ME	Mean	LB	HB	ME	2005 vs 2010	2010 vs 2014	2014 vs 2016	2005 vs 2016
Poverty	−0.110	−0.124	−0.096	0.014	−0.147	−0.161	−0.133	0.014	−0.017	− 0.022	−0.011	0.006	−0.086	− 0.096	−0.075	0.010	−0.671	−11.268	−6.664	0.021
Gender	0.005	−0.012	0.022	0.017	−0.020	−0.035	− 0.005	0.015	− 0.002	−0.009	0.004	0.006	−0.004	−0.012	0.005	0.009	0.435	0.327	1.825	1.336
Education	−0.010	− 0.026	0.006	0.016	−0.032	− 0.047	−0.017	0.015	−0.002	− 0.007	0.004	0.005	−0.012	− 0.020	−0.004	0.008	0.610	−1.012	0.470	1.888
Residence	−0.014	−0.037	0.009	0.023	0.010	−0.009	0.030	0.019	−0.009	−0.015	− 0.004	0.006	− 0.014	−0.025	− 0.004	0.010	0.878	0.458	1.398	1.983

Note: N is the number of observations, LB is the lower bound of the 95% CIs, HB is the higher bound of the 95% CIs, and ME represents the margins of error. Margins of error is the distance of either the lower or the higher bound 95% CI from the mean. The proportion overlap is defined as the intervals overlap between the two independent samples, expressed as a proportion of the average margin of error. According to Cumming and Finch [45], when both sample sizes are at least 10, and the margins of error do not differ by more than a factor of two, a proportion overlap less than 0.5 indicates a significant statistical relationship at the 0.05 level (*p* < 0.05)

The adjusted absolute weighted mean difference from the overall mean of the district-level measures was higher than zero in all of the four survey years (0.017 in 2005, 0.016 in 2010, 0.003 in 2014, and 0.010 in 2016) (Table 3). Over time, districts such as Nyamasheke and Nyamagabe had high percentages of HCHS, and Nyarugenge and Kicukiro had low percentages of HCHS. The percentage of HCHS in the district with the highest value in 2014 (2.3% in Nyamasheke) was lower than that in the district with the lowest value in 2005 (2.7% in Nyarugenge) (Additional file 1: Table S8). The adjusted absolute weighted mean difference from the overall mean decreased by 38.74% from 2005 to 2016 (Table 3). When using the relative inequality measures, the district-level trend from 2005 to 2014 remained unchanged, while that from 2014 to 2016 was reversed (Additional file 1: Table S4). In addition, among the 30 districts, Rusizi (− 60.22%) demonstrated the largest decline in the adjusted percentage of HCHS from 2005 to 2016, followed by Kamonyi (− 58.97%) and Nyaruguru (− 57.35%). The district of Nyamasheke (− 17.39%) showed the smallest decrease in the adjusted percentage of HCHS (Additional file 1: Table S8).

The adjusted national levels of medical care utilization and HCHS in the four years by different socio-demographic groups are presented in the Additional file 1: Table S6. The adjusted district-level mean of medical care utilization and HCHS in the four years is presented in the Additional file 1: Tables S7–S8, Figures S3–S4, respectively.

## Discussion

This study has two salient findings. First, at the national level, in each of the years 2005, 2010, 2014, and 2016, after controlling for other factors, significant inequalities persisted in Rwanda by poverty and education for medical care utilization and by poverty for HCHS when using either the absolute or the relative inequality measures. In all four years, non-poor residents used more medical care and incurred less HCHS than poor residents; individuals with head of household who had received no schooling used less medical care compared to their counterparts who had an educated head of household. At the district level, the estimates suggest the existence of sub-national inequalities in all of the four years under review. Second, from 2005 to 2016, at the national level, there was no significant change in the inequalities in medical care utilization by the four dimensions, except for the inequality by gender that showed a small reduction. The inequalities by poverty for HCHS were significantly reduced. At the district level, the absolute inequality in medical care utilization decreased by 27%, and in HCHS it decreased by 39%. Most of the results are robust when using relative measures.

Our study suggests that inequalities in medical care utilization were mainly derived from poverty and education, and that differences in gender or place of residence was either very small or no longer statistically significant after adjusting for other factors. This is consistent with previous studies in Rwanda [17, 24] as well as in other countries (e.g. Burkina Faso, India, and China) [36, 37, 48]. A household’s poverty level and the education level of its head influenced the likelihood of an individual seeking care when they fell ill. Analysis of the data reveal that, there were 52.8% (2005), 42.9% (2010), 35.3% (2014), and 33.2% (2016) of the sampled households living in poverty and 31.6% (2005), 28.6% (2010), 28.1% (2014), and 26.2% (2016) having an illiterate person as the head of the household (Additional file 1: Table S2), which may explain the persistence of inequality in medical care utilization.

Even in 2016, the year that achieved the highest percentage of medical care utilization when in need, the average likelihood of using needed care was only 58.9% for the non-poverty group, 57.2% for the residents with a literate head of household, and 57.8% for the residents living in urban areas. This finding is very concerning and suggests that more activities could be taken to promote service utilization.

Plausible explanations for the significant reduction of the inequality in HCHS by poverty from 2005 to 2016 could be the following. *Mutuelles*, the community-based health insurance in rural Rwanda, provided a pro-poor benefit package and contributed to the reduction of the inequality of HCHS by poverty after 2011. In both 2005 and 2010, households, regardless of their poverty status, had to pay the same amount of annual premium and copayments [49]. For example, in 2010, *Mutuelles* enrollees had to pay US$ 0.36 per outpatient visit and 10% of any hospitalization fee. For those households living under the poverty line (US$0.32 per person per day), these copayments may have prevented those residents living in poverty from seeking medical care even with their enrollment [17]. Since 2011, the Government of Rwanda has carried out a full subsidy for premiums and copayments for the poorest members of the country’s population [50]. This could have a positive effect on mitigating the inequalities in HCHS by poverty status because the results of this study suggest that health insurance enrollment had a negative association with the likelihood of incurring HCHS.

This study is subject to some potential limitations. First, factors (e.g. preferences and satisfaction of services) that might contribute to the estimation of adjusted inequalities in medical care utilization and HCHS were not included in our analysis due to the unavailability of data. Second, due to survey instrument variations across the four survey years, we were not able to include other medical services (e.g. inpatient care) in our analysis. Third, also due to the survey instrument variations across the four survey years, medical care utilization was recorded with a recall period of two weeks in EICV 2 and EICV 3, and with a recall period of four weeks in EICV 4 and EICV 5. Different recall periods could affect the measurements of medical care utilization and hence alter the over-time comparisons, as suggested in our previous study [30, 51]. Fourth, the data were self-reported and may be subject to measurement errors, such as recall bias [30, 51].

## Conclusions

Rwanda has made remarkable progress in improving equal access to medical care and financial risk protection for vulnerable people. In spite of these achievements, the existence of significant health inequalities at the national and sub-national levels is still one of the major challenges faced by the Government of Rwanda. To eliminate the magnitude of the national inequalities in medical care utilization, policy targets should focus on delivering medical care to poor households or households headed by illiterate individuals.

## Additional file

Additional file 1: **Box S1.** Sampling and implementation processes of the Integrated Living Conditions Survey (EICV). **Box S2.** Measurement of household catastrophic health spending (HCHS). **Box S3.** Methods of obtaining proportion overlap using Cumming and Finch’s “rule of thumb”. **Table S1.** Summary statistics for variables used in regression models on medical care utilization. **Table S2.** Summary statistics for variables used in regression models on HCHS. **Table S3.** Significance testing of the trends of relative inequality in medical care utilization from 2005 to 2016 using Cumming and Finch’s “rule of thumb”. **Table S4.** The relative weighted difference between the district and the national means for medical care utilization and HCHS. **Table S5.** Significance testing of the difference of relative inequality in HCHS from 2005 to 2016 using Cumming and Finch’s “rule of thumb”. **Table S6.** The adjusted levels of medical care utilization and HCHS by gender, poverty, education and residence at the national level. **Table S7.** The adjusted levels and absolute difference of medical care utilization at the district level. **Table S8.** The adjusted levels and absolute difference of HCHS at the district level. **Figure S1.** The adjusted relative inequality of medical care utilization by the status of poverty, gender, education and residence. **Figure S2.** The adjusted relative inequality of HCHS by the status of poverty, gender, education and residence. **Figure S3.** The adjusted levels of medical care utilization of all districts. **Figure S4.** The adjusted levels of HCHS of all districts. (DOCX 2289 kb)

## Abbreviations

EICV: The Rwanda Integrated Living Conditions Survey
HCHS: Household catastrophic health spending
OOPS: Out-of-pocket health spending
SDGs: Sustainable Development Goals

## Footnotes

1 Adjusted absolute weighted mean from overall mean= $\Sigma \frac{Pop_i\times \mid {r}_i-r\mid }{Pop}$, where *Pop* is the national population size, *Pop*_*i*_ is the population size of district *i*, *r* is the adjusted national mean, *r*_*i*_ is the adjusted mean of district *i*.

2 The adjusted relative weighted mean from overall mean is the absolute weighted mean from the overall mean divided by the adjusted national mean
