# Supplementary material for: Assessing national and subnational inequalities in medical care utilization and financial risk protection in Rwanda
Source: Int J Equity Health. 2019 Mar 27;18:51. doi: 10.1186/s12939-019-0953-y (PMC6437855; doi:10.1186/s12939-019-0953-y)

Additional file 1

**Box S1** Sampling and implementation processes of the Integrated Living Conditions Survey (EICV).

The EICV is a nationally representative, repeated cross-sectional survey conducted about every five years starting in 2000. Each of the surveys lasted for 12 months, with the 2005 survey starting in January 2005 and ending in December 2005, the 2010 survey commenced in November 2010 and finished in October 2011, the 2014 survey conducted from October 2013 to October 2014, and the 2016 survey from October 2016 to October 2017. There were 6,410, 6,900, 14,308, 12,312 and 14,580 households in the 2000, 2005, 2010, 2014 and 2016 EICV, respectively, with households being selected based on a stratified two-stage sample design. Sample villages were selected within each stratum (district) systematically with probability proportional to size at the first sampling stage and sample households were selected from each sample village at the second stage.^1^

**Box S2** Measurement of household catastrophic health spending (HCHS).

Using the WHO’s approach,^2,3^ we defined a household as having HCHS if its annual OOPS exceeds 40% of the household’s annual capacity to pay. Capacity to pay was measured by the household’s total expenditure, excluding spending on basic subsistence needs that were calculated as the average annual food expenditure of those households whose food shares were in the 45th and 55th percentiles. The EICV includes questions about household spending on food, housing, education, durable goods, agriculture, and so on, making it possible to construct food spending (including self-made products and excluding alcohol, cigarettes, and restaurants) and total expenditure for each household. In addition, we used the OOPS information in the consumption module with a recall period of one year. To ensure the comparability of the estimates between years, we converted the value of OOPS and other expenditure-related items to the value of the Rwanda franc in 2010. For each household, the value of HCHS was “1” if the ratio of a household’s annual total OOPS to its capacity to pay was 0.4 or above, and “0” otherwise.

**Box S3** Methods of obtaining proportion overlap using Cumming and Finch’s “rule of thumb”.

Using Cumming and Finch’s “rule of thumb”,^4^ We obtained proportion overlap using Equation 2.1 or 2.2 as follows, depending on the size of health inequality estimate in various survey years.

If the health inequality estimate in 2005 is lower than that in 2016, then

$Proportion overlap \left( Utilization/HCHS \right)=\frac{{95\% CI upper bound}_{2005}-{95\% CI lower bound}_{2016}}{({Margin of error}_{2005}+{Margin of error}_{2016})/2}$ **(2.1)**

Or, if the health inequality estimate in 2005 is higher than that in 2016, then

$Proportion overlap \left( Utilization/HCHS \right)=\frac{{95\% CI upper bound}_{2016}-{95\% CI lower bound}_{2005}}{({Margin of error}_{2005}+{Margin of error}_{2016})/2}$ **(2.2)**

**References**

1. Rwanda National Institute of Statistics. Integrated living conditions survey (2000, 2005, 2010, 2014, and 2016). <http://www.statistics.gov.rw/survey/integrated-household-living-conditions-survey-eicv>. Accessed 16 Jan 2019.
2. Xu K, Evans DB, Kawabata K, et al. Household catastrophic health expenditure: a multicountry analysis. Lancet. 2003; 362: 111–17.
3. Xu K, Evans D, Carrin G, et al. Protecting households from catastrophic health spending. Health Aff (Millwood). 2007; 26: 972–83.
4. Cumming G, Finch S. Inference by eye: confidence intervals and how to read pictures of data. Am Psychol. 2005;60(2):170-80

**Table S1** Summary statistics for variables used in regression models on medical care utilization.

|  | 2005 | | | 2010 | | | 2014 | | | 2016 | | |
| --- | --- | --- | --- | --- | --- | --- | --- | --- | --- | --- | --- | --- |
|  | N | Mean | SD | N | Mean | SD | N | Mean | SD | N | Mean | SD |
| Medical care utilization | 6,737 | 0.318 | 0.466 | 11,944 | 0.391 | 0.488 | 16,807 | 0.553 | 0.497 | 21,150 | 0.567 | 0.495 |
| Poverty | 6,737 | 0.540 | 0.498 | 11,944 | 0.440 | 0.496 | 16,807 | 0.392 | 0.488 | 21,150 | 0.401 | 0.490 |
| Female | 6,737 | 0.560 | 0.496 | 11,944 | 0.584 | 0.493 | 16,807 | 0.573 | 0.495 | 21,150 | 0.562 | 0.496 |
| Head: no education | 6,737 | 0.307 | 0.461 | 11,940 | 0.275 | 0.446 | 16,807 | 0.288 | 0.453 | 21,150 | 0.277 | 0.448 |
| Rural | 6,737 | 0.787 | 0.409 | 11,944 | 0.852 | 0.355 | 16,807 | 0.867 | 0.340 | 21,150 | 0.870 | 0.336 |
| Age group <30 | 6,737 | 0.661 | 0.473 | 11,944 | 0.614 | 0.487 | 16,807 | 0.616 | 0.486 | 21,150 | 0.631 | 0.482 |
| Age group 30–50 | 6,737 | 0.202 | 0.401 | 11,944 | 0.205 | 0.403 | 16,807 | 0.202 | 0.402 | 21,150 | 0.205 | 0.404 |
| Age group >50 | 6,737 | 0.137 | 0.344 | 11,944 | 0.182 | 0.386 | 16,807 | 0.182 | 0.386 | 21,150 | 0.164 | 0.370 |
| Disability | 6,737 | 0.063 | 0.243 | 11,944 | 0.078 | 0.269 | 16,807 | 0.063 | 0.244 | 21,150 | 0.049 | 0.217 |
| Household size | 6,737 | 5.701 | 2.386 | 11,944 | 5.371 | 2.198 | 16,807 | 5.172 | 2.123 | 21,150 | 5.151 | 2.088 |
| Health insurance (individual) | 6,737 | 0.398 | 0.490 | 11,944 | 0.679 | 0.467 | 16,807 | 0.706 | 0.456 | 21,150 | 0.725 | 0.446 |
| Travel time to health center (>0.5 hour) | 6,737 | 0.720 | 0.449 | 11,944 | 0.696 | 0.460 | 16,807 | 0.625 | 0.484 | 21,150 | 0.590 | 0.492 |

**Table S2** Summary statistics for variables used in regression models on HCHS.

|  | 2005 | | | 2010 | | | 2014 | | | 2016 | | |
| --- | --- | --- | --- | --- | --- | --- | --- | --- | --- | --- | --- | --- |
|  | N | Mean | SD | N | Mean | SD | N | Mean | SD | N | Mean | SD |
| HCHS | 6,639 | 0.084 | 0.278 | 11,335 | 0.090 | 0.288 | 14,125 | 0.018 | 0.134 | 14,548 | 0.046 | 0.210 |
| Poverty | 6,639 | 0.528 | 0.499 | 11,335 | 0.429 | 0.495 | 14,125 | 0.353 | 0.478 | 14,548 | 0.332 | 0.471 |
| Head: female | 6,639 | 0.287 | 0.452 | 11,335 | 0.291 | 0.454 | 14,125 | 0.259 | 0.438 | 14,548 | 0.255 | 0.436 |
| Head: no education | 6,639 | 0.316 | 0.465 | 11,333 | 0.286 | 0.452 | 14,125 | 0.281 | 0.449 | 14,548 | 0.262 | 0.440 |
| Rural | 6,639 | 0.783 | 0.412 | 11,335 | 0.861 | 0.346 | 14,125 | 0.850 | 0.357 | 14,548 | 0.827 | 0.378 |
| Head: age group <30 | 6,639 | 0.223 | 0.416 | 11,335 | 0.213 | 0.410 | 14,125 | 0.202 | 0.401 | 14,548 | 0.189 | 0.391 |
| Head: age group 30–50 | 6,639 | 0.473 | 0.499 | 11,335 | 0.443 | 0.497 | 14,125 | 0.441 | 0.496 | 14,548 | 0.467 | 0.499 |
| Head: age group >50 | 6,639 | 0.304 | 0.460 | 11,335 | 0.344 | 0.475 | 14,125 | 0.358 | 0.479 | 14,548 | 0.344 | 0.475 |
| Household having child | 6,639 | 0.590 | 0.492 | 11,335 | 0.566 | 0.496 | 14,125 | 0.540 | 0.498 | 14,548 | 0.524 | 0.499 |
| Household having disabled people | 6,639 | 0.162 | 0.369 | 11,335 | 0.182 | 0.386 | 14,125 | 0.163 | 0.369 | 14,548 | 0.147 | 0.354 |
| Household size | 6,639 | 4.966 | 2.294 | 11,335 | 4.645 | 2.136 | 14,125 | 4.615 | 2.094 | 14,548 | 4.418 | 2.115 |
| Health insurance (household) | 6,639 | 0.437 | 0.496 | 11,335 | 0.656 | 0.475 | 14,125 | 0.713 | 0.452 | 14,548 | 0.753 | 0.432 |
| Travel time to health center (>0.5 hour) | 6,639 | 0.706 | 0.456 | 11,335 | 0.707 | 0.455 | 14,125 | 0.610 | 0.488 | 14,548 | 0.567 | 0.495 |

**Table S3** Significance testing of the trends of relative inequality in medical care utilization from 2005 to 2016 using Cumming and Finch’s “rule of thumb”.

|  | 2005 | | | | 2010 | | | | 2014 | | | | 2016 | | | | Proportion overlap | | | |
| --- | --- | --- | --- | --- | --- | --- | --- | --- | --- | --- | --- | --- | --- | --- | --- | --- | --- | --- | --- | --- |
|  | Mean | LB | HB | ME | Mean | LB | HB | ME | Mean | LB | HB | ME | Mean | LB | HB | ME | 2005  vs  2010 | 2010  vs  2014 | 2014  vs  2016 | 2005  vs  2016 |
| Poverty | 1.303 | 1.202 | 1.403 | 0.101 | 1.260 | 1.194 | 1.327 | 0.066 | 1.153 | 1.119 | 1.187 | 0.034 | 1.128 | 1.098 | 1.158 | 0.030 | 1.493 | -0.133 | 1.216 | -0.666 |
| Gender | 1.066 | 0.989 | 1.143 | 0.077 | 0.991 | 0.943 | 1.039 | 0.048 | 0.994 | 0.968 | 1.021 | 0.026 | 0.982 | 0.957 | 1.006 | 0.024 | 0.803 | 1.913 | 1.498 | 0.337 |
| Education | 1.092 | 1.000 | 1.184 | 0.092 | 1.064 | 1.000 | 1.128 | 0.064 | 1.085 | 1.052 | 1.119 | 0.034 | 1.044 | 1.016 | 1.073 | 0.029 | 1.650 | 1.567 | 0.685 | 1.218 |
| Residence | 1.052 | 0.954 | 1.149 | 0.097 | 0.971 | 0.908 | 1.035 | 0.063 | 1.041 | 0.999 | 1.083 | 0.042 | 0.978 | 0.934 | 1.023 | 0.045 | 0.874 | 0.685 | 0.555 | 0.965 |

Note: LB is the lower bound of the 95% CIs, HB is the higher bound of the 95% CIs, and ME refers to the margins of error. Margins of error is the distance of either the lower or the higher bound 95% CI from the mean. The proportion overlap is defined as the intervals overlap between the two independent samples, expressed as a proportion of the average margin of error. According to Cumming and Finch [45], when both sample sizes are at least 10, and the margins of error do not differ by more than a factor of two, a proportion overlap less than 0.5 indicates a significant statistical relationship at the 0.05 level (p<0.05)

**Table S4** The relative weighted difference between the district and the national means for medical care utilization and HCHS.

|  | 2005 | 2010 | 2014 | 2016 | Percent change from 2005 to 2016 |
| --- | --- | --- | --- | --- | --- |
| Medical care utilization | 0.089 | 0.104 | 0.059 | 0.035 | -59.89% |
| HCHS | 0.191 | 0.180 | 0.142 | 0.227 | 18.76% |

**Table S5** Significance testing of the difference of relative inequality in HCHS from 2005 to 2016 using Cumming and Finch’s “rule of thumb”.

|  | 2005 | | | | 2010 | | | | 2014 | | | | 2016 | | | | Proportion overlap | | | |
| --- | --- | --- | --- | --- | --- | --- | --- | --- | --- | --- | --- | --- | --- | --- | --- | --- | --- | --- | --- | --- |
|  | Mean | LB | HB | ME | Mean | LB | HB | ME | Mean | LB | HB | ME | Mean | LB | HB | ME | 2005  vs  2010 | 2010  vs  2014 | 2014  vs  2016 | 2005  vs  2016 |
| Poverty | 0.178 | 0.126 | 0.230 | 0.052 | 0.177 | 0.147 | 0.208 | 0.031 | 0.423 | 0.312 | 0.535 | 0.112 | 0.164 | 0.131 | 0.196 | 0.032 | 0.874 | -1.449 | -1.602 | 1.653 |
| Gender | 1.056 | 0.850 | 1.263 | 0.206 | 0.813 | 0.694 | 0.933 | 0.119 | 0.882 | 0.592 | 1.173 | 0.291 | 0.923 | 0.752 | 1.094 | 0.171 | 0.874 | 1.665 | 1.823 | 1.292 |
| Education | 0.890 | 0.731 | 1.049 | 0.159 | 0.722 | 0.618 | 0.826 | 0.104 | 0.920 | 0.659 | 1.181 | 0.261 | 0.777 | 0.648 | 0.905 | 0.128 | 0.874 | 0.916 | 1.266 | 1.212 |
| Residence | 0.846 | 0.591 | 1.100 | 0.254 | 1.110 | 0.895 | 1.325 | 0.215 | 0.522 | 0.254 | 0.790 | 0.268 | 0.696 | 0.478 | 0.913 | 0.217 | 0.873 | -0.438 | 1.283 | 1.364 |

Note: LB is the lower bound of the 95% CIs, HB is the higher bound of the 95% CIs, and ME refers to the margins of error. Margins of error is the distance of either the lower or the higher bound 95% CI from the mean. The proportion overlap is defined as the intervals overlap between the two independent samples, expressed as a proportion of the average margin of error. According to Cumming and Finch [45], when both sample sizes are at least 10, and the margins of error do not differ by more than a factor of two, a proportion overlap less than 0.5 indicates a significant statistical relationship at the 0.05 level (p<0.05)

**Table S6** The adjusted levels of medical care utilization and HCHS by gender, poverty, education, and residence at the national level.

|  | 2005 | 2010 | 2014 | 2016 |
| --- | --- | --- | --- | --- |
|  | Mean (95% CI) | Mean (95% CI) | Mean (95% CI) | Mean (95% CI) |
| *(1) Medical care utilization when reporting illnesses* | | |  |  |
| *By poverty* | | |  |  |
| Non-poverty | 0.361(0.343,0.380) | 0.435(0.422,0.448) | 0.589(0.580,0.598) | 0.595(0.586,0.604) |
| Poverty | 0.277(0.262,0.293) | 0.345(0.331,0.360) | 0.511(0.498,0.523) | 0.528(0.516,0.539) |
| *By gender* | | |  |  |
| Male | 0.327(0.310,0.344) | 0.396(0.381,0.411) | 0.557(0.546,0.568) | 0.563(0.553,0.574) |
| Female | 0.307(0.292,0.322) | 0.399(0.387,0.412) | 0.560(0.551,0.570) | 0.574(0.565,0.583) |
| *By education* | | |  |  |
| Schooling | 0.324(0.310,0.338) | 0.404(0.394,0.415) | 0.572(0.563,0.580) | 0.576(0.568,0.584) |
| No Schooling | 0.297(0.276,0.318) | 0.380(0.360,0.400) | 0.527(0.513,0.541) | 0.551(0.538,0.564) |
| *By region* | | |  |  |
| Urban | 0.329(0.302,0.356) | 0.388(0.365,0.411) | 0.578(0.556,0.600) | 0.558(0.534,0.583) |
| Rural | 0.313(0.300,0.326) | 0.400(0.389,0.410) | 0.555(0.548,0.563) | 0.571(0.564,0.578) |
| *(2) % of households with catastrophic health spending* | | |  |  |
| *By poverty* | | |  |  |
| Non-poverty | 0.024(0.017,0.030) | 0.032(0.027,0.037) | 0.012(0.010,0.014) | 0.017(0.014,0.020) |
| Poverty | 0.134(0.122,0.146) | 0.179(0.166,0.192) | 0.029(0.024,0.034) | 0.103(0.093,0.113) |
| *By gender* | | |  |  |
| Male | 0.089(0.081,0.098) | 0.087(0.080,0.094) | 0.017(0.015,0.020) | 0.043(0.039,0.048) |
| Female | 0.085(0.071,0.098) | 0.107(0.094,0.119) | 0.020(0.015,0.025) | 0.047(0.040,0.054) |
| *By education* | | |  |  |
| Schooling | 0.084(0.075,0.093) | 0.083(0.076,0.090) | 0.018(0.015,0.020) | 0.041(0.037,0.045) |
| No Schooling | 0.094(0.082,0.107) | 0.115(0.102,0.127) | 0.019(0.015,0.023) | 0.052(0.046,0.059) |
| *By region* | | |  |  |
| Urban | 0.075(0.054,0.097) | 0.102(0.084,0.120) | 0.010(0.005,0.015) | 0.032(0.023,0.042) |
| Rural | 0.089(0.082,0.097) | 0.092(0.086,0.098) | 0.019(0.017,0.022) | 0.046(0.042,0.050) |

**Table S7** The adjusted levels and absolute difference of medical care utilization at the district level.

|  | District | 2005 |  | 2010 | | 2014 | | 2016 | | Percent change from 2005 to 2016 |
| --- | --- | --- | --- | --- | --- | --- | --- | --- | --- | --- |
|  |  | Adjusted mean | Adjusted absolute difference | Adjusted mean | Adjusted absolute difference | Adjusted mean | Adjusted absolute difference | Adjusted mean | Adjusted absolute difference | Adjusted mean |
| 1 | Nyaruguru | 0.229 | -0.083 | 0.338 | -0.055 | 0.496 | -0.059 | 0.545 | -0.022 | 137.99% |
| 2 | Gisagara | 0.250 | -0.062 | 0.317 | -0.076 | 0.517 | -0.038 | 0.549 | -0.019 | 119.60% |
| 3 | Burera | 0.283 | -0.029 | 0.437 | 0.044 | 0.597 | 0.041 | 0.595 | 0.027 | 110.25% |
| 4 | Gicumbi | 0.313 | 0.001 | 0.426 | 0.033 | 0.568 | 0.013 | 0.617 | 0.049 | 97.12% |
| 5 | Ruhango | 0.290 | -0.022 | 0.337 | -0.056 | 0.532 | -0.024 | 0.569 | 0.002 | 96.21% |
| 6 | Nyamagabe | 0.273 | -0.039 | 0.271 | -0.122 | 0.523 | -0.032 | 0.533 | -0.035 | 95.24% |
| 7 | Kirehe | 0.311 | -0.001 | 0.427 | 0.034 | 0.590 | 0.035 | 0.601 | 0.033 | 93.25% |
| 8 | Kamonyi | 0.295 | -0.017 | 0.419 | 0.026 | 0.568 | 0.013 | 0.569 | 0.001 | 92.88% |
| 9 | Ngororero | 0.279 | -0.032 | 0.382 | -0.011 | 0.532 | -0.023 | 0.536 | -0.032 | 92.11% |
| 10 | Huye | 0.286 | -0.026 | 0.365 | -0.028 | 0.590 | 0.035 | 0.549 | -0.019 | 91.96% |
| 11 | Gakenke | 0.321 | 0.009 | 0.422 | 0.029 | 0.599 | 0.043 | 0.608 | 0.040 | 89.41% |
| 12 | Bugesera | 0.311 | -0.001 | 0.384 | -0.009 | 0.566 | 0.011 | 0.581 | 0.013 | 86.82% |
| 13 | Rusizi | 0.303 | -0.009 | 0.423 | 0.030 | 0.509 | -0.047 | 0.559 | -0.009 | 84.49% |
| 14 | Musanze | 0.329 | 0.017 | 0.456 | 0.063 | 0.579 | 0.024 | 0.605 | 0.037 | 83.89% |
| 15 | Nyamasheke | 0.322 | 0.010 | 0.428 | 0.035 | 0.512 | -0.043 | 0.588 | 0.020 | 82.61% |
| 16 | Ngoma | 0.318 | 0.006 | 0.425 | 0.032 | 0.561 | 0.006 | 0.577 | 0.009 | 81.45% |
| 17 | Karongi | 0.304 | -0.008 | 0.373 | -0.020 | 0.504 | -0.052 | 0.547 | -0.021 | 79.93% |
| 18 | Rwamagana | 0.318 | 0.006 | 0.426 | 0.033 | 0.594 | 0.038 | 0.562 | -0.006 | 76.73% |
| 19 | Gatsibo | 0.327 | 0.015 | 0.369 | -0.024 | 0.585 | 0.030 | 0.576 | 0.008 | 76.15% |
| 20 | Kayonza | 0.326 | 0.014 | 0.431 | 0.038 | 0.601 | 0.046 | 0.574 | 0.006 | 76.07% |
| 21 | Nyanza | 0.284 | -0.028 | 0.292 | -0.101 | 0.523 | -0.032 | 0.499 | -0.069 | 75.70% |
| 22 | Muhanga | 0.325 | 0.013 | 0.389 | -0.004 | 0.565 | 0.009 | 0.571 | 0.003 | 75.69% |
| 23 | Rutsiro | 0.326 | 0.014 | 0.381 | -0.012 | 0.497 | -0.058 | 0.562 | -0.006 | 72.39% |
| 24 | Rubavu | 0.323 | 0.011 | 0.372 | -0.021 | 0.525 | -0.030 | 0.547 | -0.021 | 69.35% |
| 25 | Nyabihu | 0.332 | 0.020 | 0.388 | -0.005 | 0.545 | -0.011 | 0.562 | -0.006 | 69.28% |
| 26 | Rulindo | 0.340 | 0.028 | 0.364 | -0.028 | 0.569 | 0.014 | 0.569 | 0.001 | 67.35% |
| 27 | Nyagatare | 0.330 | 0.018 | 0.427 | 0.034 | 0.586 | 0.031 | 0.533 | -0.035 | 61.52% |
| 28 | Gasabo | 0.371 | 0.059 | 0.433 | 0.040 | 0.557 | 0.002 | 0.593 | 0.025 | 59.84% |
| 29 | Kicukiro | 0.389 | 0.077 | 0.477 | 0.084 | 0.625 | 0.069 | 0.577 | 0.009 | 48.33% |
| 30 | Nyarugenge | 0.428 | 0.116 | 0.483 | 0.090 | 0.613 | 0.058 | 0.602 | 0.035 | 40.65% |

**Table S8** The adjusted levels and absolute difference of HCHS at the district level.

|  | District | 2005 |  | 2010 | | 2014 | | 2016 | | Percent change from 2005 to 2016 |
| --- | --- | --- | --- | --- | --- | --- | --- | --- | --- | --- |
|  |  | Adjusted mean | Adjusted absolute difference | Adjusted mean | Adjusted absolute difference | Adjusted mean | Adjusted absolute difference | Adjusted mean | Adjusted absolute difference | Adjusted mean |
| 1 | Rusizi | 0.093 | 0.005 | 0.077 | -0.013 | 0.018 | 0.000 | 0.037 | -0.008 | -60.22% |
| 2 | Kamonyi | 0.078 | -0.010 | 0.081 | -0.009 | 0.017 | -0.002 | 0.032 | -0.013 | -58.97% |
| 3 | Nyaruguru | 0.136 | 0.049 | 0.109 | 0.018 | 0.022 | 0.004 | 0.058 | 0.012 | -57.35% |
| 4 | Huye | 0.100 | 0.012 | 0.082 | -0.009 | 0.016 | -0.002 | 0.044 | -0.001 | -56.00% |
| 5 | Gicumbi | 0.093 | 0.006 | 0.099 | 0.009 | 0.021 | 0.003 | 0.041 | -0.004 | -55.91% |
| 6 | Gisagara | 0.131 | 0.044 | 0.108 | 0.018 | 0.023 | 0.005 | 0.061 | 0.015 | -53.44% |
| 7 | Nyamagabe | 0.117 | 0.029 | 0.136 | 0.045 | 0.020 | 0.002 | 0.055 | 0.010 | -52.99% |
| 8 | Rubavu | 0.085 | -0.002 | 0.078 | -0.013 | 0.016 | -0.002 | 0.040 | -0.006 | -52.94% |
| 9 | Kayonza | 0.073 | -0.015 | 0.088 | -0.003 | 0.016 | -0.003 | 0.036 | -0.009 | -50.68% |
| 10 | Ruhango | 0.099 | 0.011 | 0.120 | 0.029 | 0.020 | 0.002 | 0.049 | 0.004 | -50.51% |
| 11 | Rwamagana | 0.062 | -0.026 | 0.073 | -0.018 | 0.017 | -0.001 | 0.031 | -0.014 | -50.00% |
| 12 | Kicukiro | 0.037 | -0.051 | 0.045 | -0.046 | 0.009 | -0.009 | 0.019 | -0.026 | -48.65% |
| 13 | Bugesera | 0.091 | 0.003 | 0.099 | 0.008 | 0.018 | 0.000 | 0.047 | 0.001 | -48.35% |
| 14 | Burera | 0.108 | 0.021 | 0.084 | -0.006 | 0.020 | 0.002 | 0.056 | 0.011 | -48.15% |
| 15 | Kirehe | 0.105 | 0.017 | 0.097 | 0.006 | 0.018 | 0.000 | 0.055 | 0.010 | -47.62% |
| 16 | Gasabo | 0.047 | -0.040 | 0.069 | -0.021 | 0.012 | -0.006 | 0.025 | -0.020 | -46.81% |
| 17 | Gakenke | 0.088 | 0.000 | 0.104 | 0.014 | 0.019 | 0.001 | 0.047 | 0.001 | -46.59% |
| 18 | Gatsibo | 0.090 | 0.002 | 0.091 | 0.000 | 0.020 | 0.002 | 0.049 | 0.003 | -45.56% |
| 19 | Nyanza | 0.097 | 0.010 | 0.104 | 0.013 | 0.019 | 0.001 | 0.054 | 0.009 | -44.33% |
| 20 | Nyagatare | 0.083 | -0.004 | 0.088 | -0.003 | 0.018 | 0.000 | 0.047 | 0.002 | -43.37% |
| 21 | Ngororero | 0.096 | 0.008 | 0.101 | 0.011 | 0.021 | 0.003 | 0.056 | 0.011 | -41.67% |
| 22 | Muhanga | 0.074 | -0.014 | 0.098 | 0.008 | 0.018 | 0.000 | 0.044 | -0.002 | -40.54% |
| 23 | Musanze | 0.084 | -0.004 | 0.050 | -0.040 | 0.016 | -0.002 | 0.051 | 0.005 | -39.29% |
| 24 | Ngoma | 0.078 | -0.010 | 0.096 | 0.005 | 0.019 | 0.001 | 0.048 | 0.002 | -38.46% |
| 25 | Rutsiro | 0.094 | 0.007 | 0.108 | 0.018 | 0.022 | 0.004 | 0.058 | 0.013 | -38.30% |
| 26 | Karongi | 0.087 | -0.001 | 0.107 | 0.017 | 0.022 | 0.004 | 0.058 | 0.012 | -33.33% |
| 27 | Nyabihu | 0.079 | -0.009 | 0.066 | -0.024 | 0.017 | -0.002 | 0.053 | 0.008 | -32.91% |
| 28 | Rulindo | 0.077 | -0.011 | 0.090 | 0.000 | 0.021 | 0.002 | 0.060 | 0.015 | -22.08% |
| 29 | Nyarugenge | 0.027 | -0.061 | 0.051 | -0.040 | 0.011 | -0.008 | 0.022 | -0.024 | -18.52% |
| 30 | Nyamasheke | 0.092 | 0.004 | 0.113 | 0.023 | 0.023 | 0.005 | 0.076 | 0.031 | -17.39% |

**Figure S1** The adjusted relative inequality of medical care utilization by the status of poverty, gender, education, and residence.


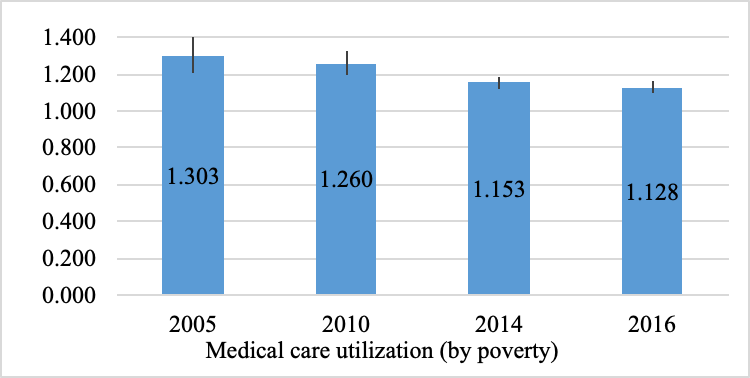

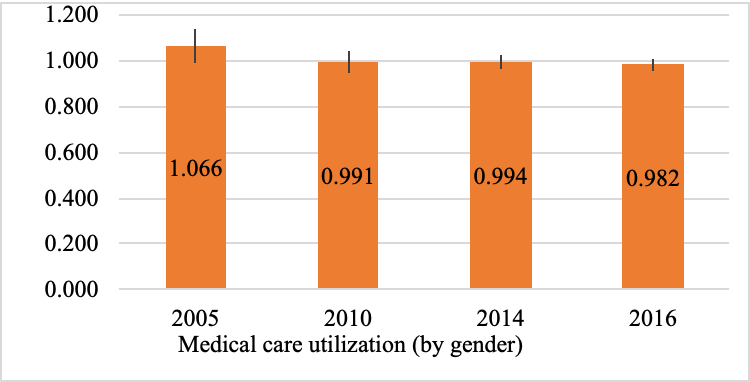

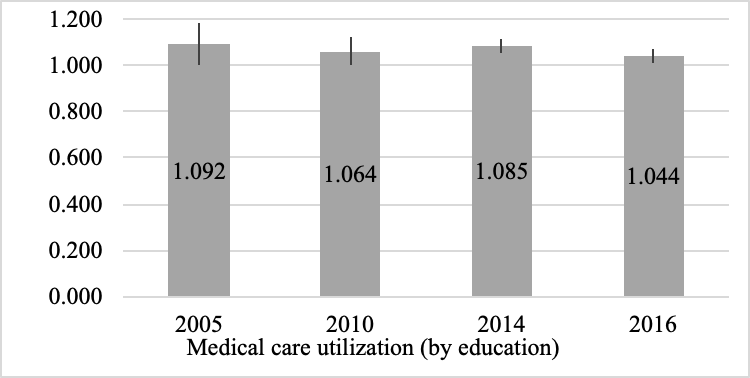

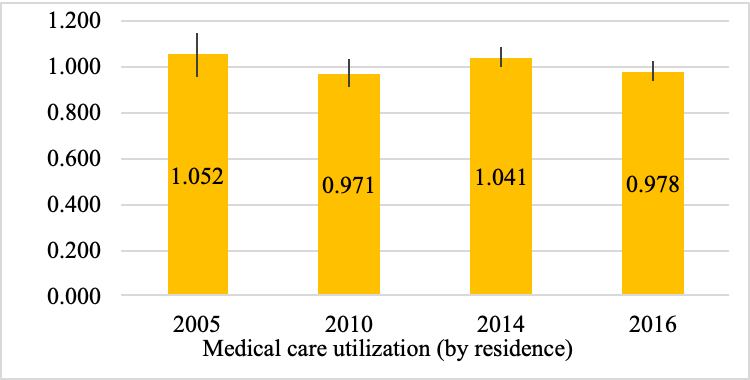


**Figure S2** The adjusted relative inequality of HCHS by the status of poverty, gender, education, and residence.


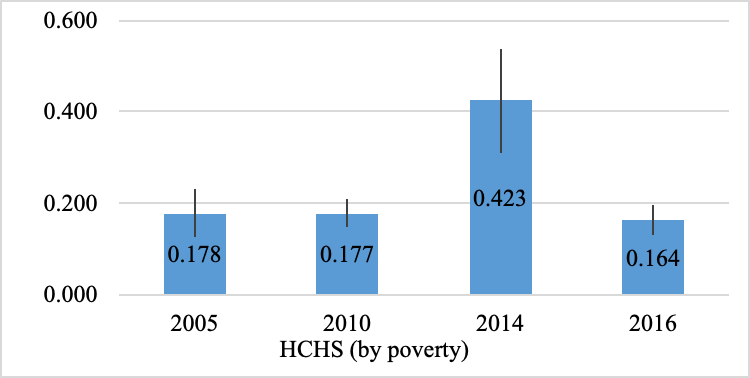

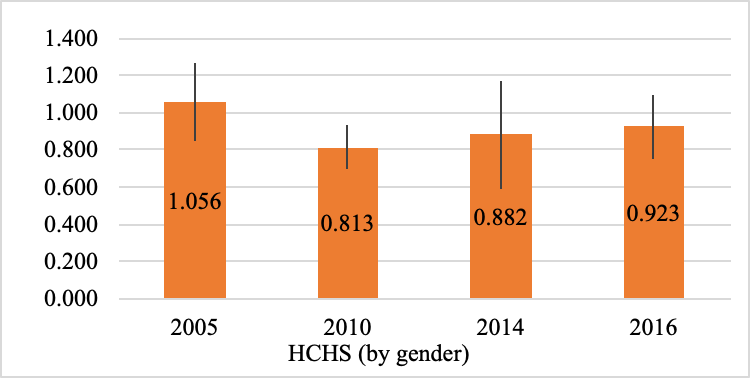

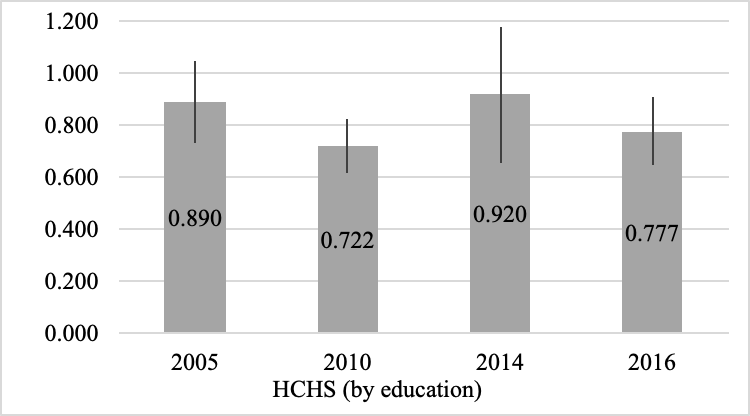

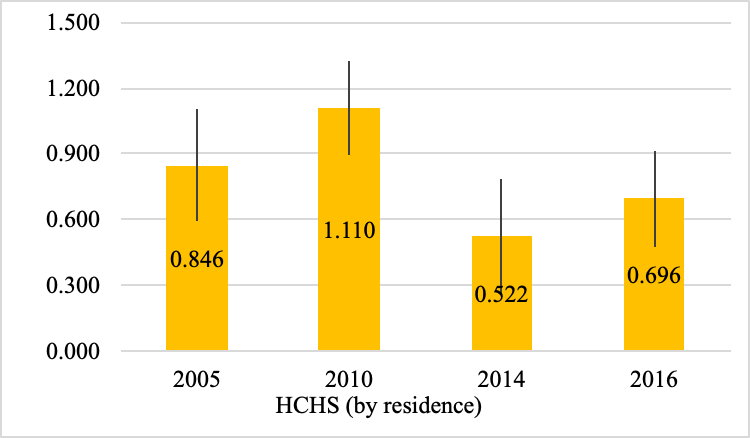


**Figure S3** The adjusted levels of medical care utilization of all districts.


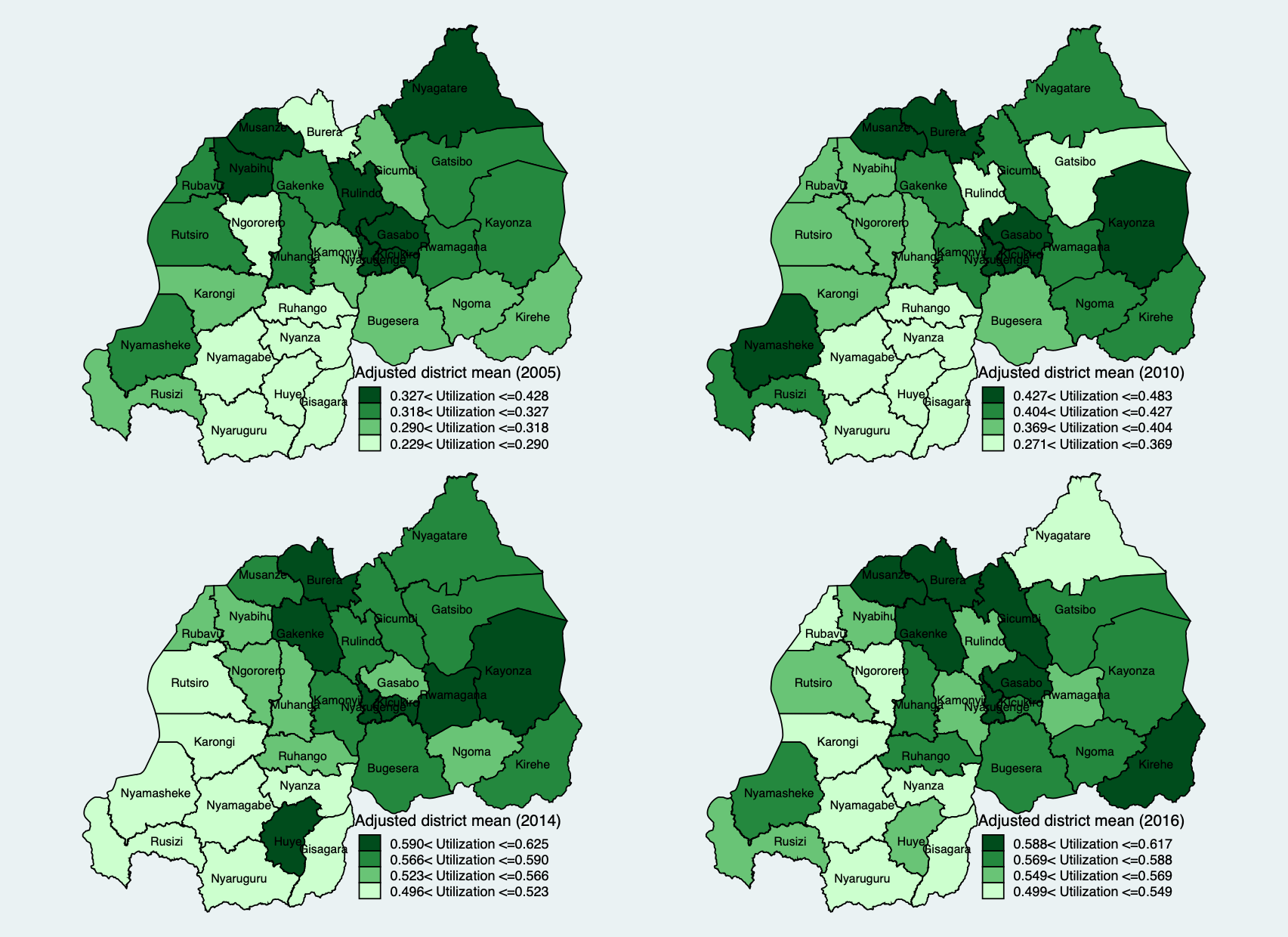


**Figure S4** The adjusted levels of HCHS of all districts.


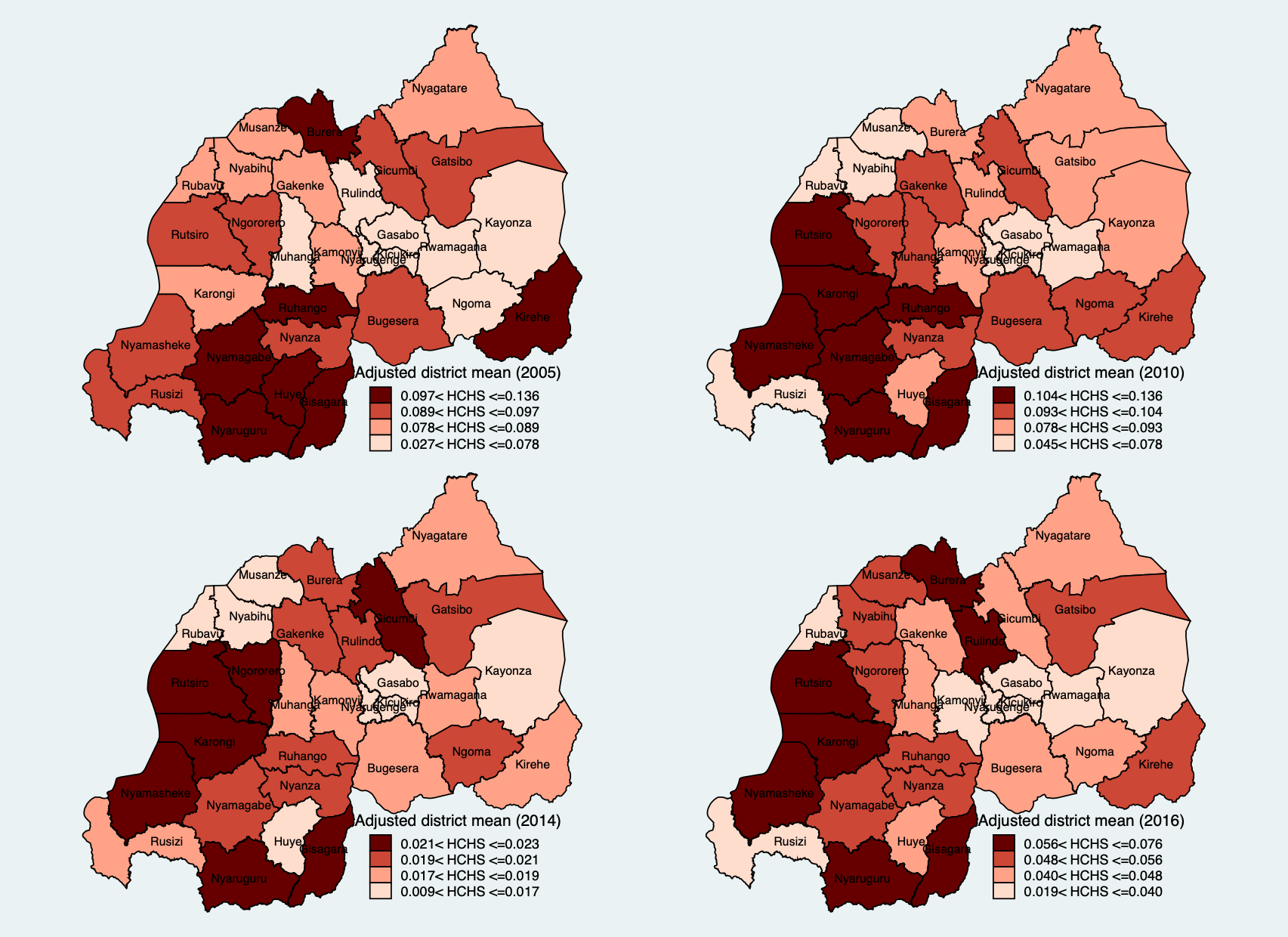

Supplement: Supplementary file 1 — Box S1. Sampling and implementation processes of the Integrated Living Conditions Survey (EICV). Box S2. Measurement of household catastrophic health spending (HCHS). Box S3. Methods of obtaining proportion overlap using Cumming and Finch’s “rule of thumb”. Table S1. Summary statistics for variables used in regression models on medical care utilization. Table S2. Summary statistics for variables used in regression models on HCHS. Table S3. Significance testing of the trends of relative inequality in medical care utilization from 2005 to 2016 using Cumming and Finch’s “rule of thumb”. Table S4. The relative weighted difference between the district and the national means for medical care utilization and HCHS. Table S5. Significance testing of the difference of relative inequality in HCHS from 2005 to 2016 using Cumming and Finch’s “rule of thumb”. Table S6. The adjusted levels of medical care utilization and HCHS by gender, poverty, education and residence at the national level. Table S7. The adjusted levels and absolute difference of medical care utilization at the district level. Table S8. The adjusted levels and absolute difference of HCHS at the district level. Figure S1. The adjusted relative inequality of medical care utilization by the status of poverty, gender, education and residence. Figure S2. The adjusted relative inequality of HCHS by the status of poverty, gender, education and residence. Figure S3. The adjusted levels of medical care utilization of all districts. Figure S4. The adjusted levels of HCHS of all districts. (DOCX 2289 kb) [file 12939_2019_953_MOESM1_ESM.docx]
